# Establishment and Characterization of an Omasal Epithelial Cell Model Derived from Dairy Calves for the Study of Small Peptide Absorption

**DOI:** 10.1371/journal.pone.0088993

**Published:** 2014-03-14

**Authors:** Qingbiao Xu, Yueming Wu, Hongyun Liu, Yingming Xie, Xinbei Huang, Jianxin Liu

**Affiliations:** Institute of Dairy Science, College of Animal Sciences, Key Laboratory of Molecular Animal Nutrition, Ministry of Education, Zhejiang University, Hangzhou, P.R. China; French Blood Institute, France

## Abstract

The objective of this study was to establish a primary culture of omasal epithelial cells (OECs) derived from dairy calves and to characterize its function in small peptide absorption. Bovine omasal tissues were obtained from newborn Chinese Holstein calves and digested with a 2.5% trypsin solution to obtain OECs. The isolated cells were later cultured in DMEM containing 10% fetal bovine serum, 5 μg/ml insulin, 10 ng/ml epidermal growth factor, 100 U/ml penicillin, 100 μg/ml streptomycin, 50 μg/ml gentamycin and 2.5 μg/ml amphotericin B. Hematoxylin and eosin staining of omasal tissue after digestion indicated that the cultured cells originated from the epithelial strata. Pure epithelial cells displayed an epithelial cell-like morphology, similar to cobblestone, with few visible fibroblasts and were cytokeratin 18-positive according to immunocytochemical analyses. The OECs were morphologically characterized with desmosomes, tight junctions and microvilli. These cells exhibited normal growth properties, as assessed using a cell growth curve, and were stably cultured for 10 passages. The OECs expressed the peptide transporter 1 (PEPT1) mRNA and absorbed intact glycylsarcosine (Gly-Sar). The uptake of Gly-Sar by OECs was pH-dependent with an optimal pH of 5.5–6.5. Furthermore, the uptake of Gly-Sar was also time-dependent, concentration-dependent and temperature-dependent. Moreover, PEPT1 was saturated with Gly-Sar at a concentration of 2.5 mM. The uptake via PEPT1 was higher compared with that via passive route at low substrate concentrations (<1.5 mM). This result suggested that PEPT1 contributed more to total small peptide absorption at low concentrations. In addition, this uptake could be competitively inhibited by methionine-glycine. Taken together, these data suggested that PEPT1 contributes to small peptide absorption in OECs. Thus, OECs may serve as a useful culture model for the study of the absorption of small peptides in bovine omasum.

## Introduction

The forestomach of ruminants has historically served as a constructive model for the study of epithelial transport [1]. The forestomach, particularly the omasum (bible), plays an important role in the absorption of nutrients from the ingesta, such as water, volatile fatty acids, minerals, electrolytes [2], amino acids (AA) [3], [4] and small peptides [3]–[5]. Interestingly, compared to the rumen, the omasum has a stronger ability to absorb small peptides [3], [6].

The absorption and utilization of peptides in animal nutrition have been reviewed in detail elsewhere [7]. Peptides comprise a significant portion of soluble non-ammonia nitrogen in the forestomach [8] and total AA in the portal-drained viscera of the ruminant [9]. Moreover, peptides can be utilized by the mammary gland for milk protein synthesis [10], [11] and by many other tissues for nutritional and functional activities [7]. Several studies have revealed that the transport of AA in the form of peptides is more effective than AA in the free form per unit of time. In addition, it was reported that a large quantity of di- and tripeptides and a variety of peptidomimetic drugs are absorbed into gastrointestinal epithelial cells via the peptide transporter 1 (PEPT1, SLC15 family) in the apical membranes of enterocytes [7], [12].

However, many previous studies on the absorption of small peptides in the ruminant gastrointestinal tract have been performed on animals, tissue models or in cell lines [2]–[6], [8]–[10], [13]–[15]. However, most of these cell lines have lost their organ-specific function due to their differentiated status [16]. In addition, most of these studies were short-term. Thus, the establishment of a long-term culture of primary epithelial cells isolated from the forestomach of ruminant animals may provide a better model for the study of their functions. Primary culture of rumen epithelial cells obtained from a variety of animals have been previously described, such as sheep [1], [17]–[19], heifers [20] and ewes [21], although there have been no studies reporting the long-term culture and characterization of omasal epithelial cells (OECs) obtained from dairy cows and the function of peptide absorption in these cells. The omasum, which is stratified squamous tissue, consists of four epithelial strata like the rumen: stratum corneum, stratum granulosum, stratum spinosum and stratum basale [22]. However, it is difficult to isolate these epithelial cells. This study successfully isolated these cells via serial digestion using specific trypsin concentrations and digestion times. The objectives of this study were (1) to establish and characterize bovine OEC cultures and (2) to study the functions of small peptide absorption by the bovine forestomach epithelium using OECs *in vitro*.

## Materials and Methods

### Ethics Statement

This study was performed according to the National Guidelines for Experimental Animal Welfare (Ministry of Science and Technology of China, 2006) and approved by the Institutional Animal Care and Use Committee at Zhejiang University. The omasal tissues were obtained from the Fuyang Slaughter House, Hangzhou, China and we obtained permission to use these animal parts from this slaughterhouse.

### Cell Culture and Storage Media

The cell growth medium consisted of a high glucose formulation of Dulbecco's modified Eagle medium (DMEM, Gibco, USA) supplemented with 10% (v/v) fetal bovine serum (FBS) (Gibco, USA), 5 μg/ml insulin, 10 ng/ml epidermal growth factor (EGF, Sigma, USA), 100 U/ml penicillin, 100 μg/ml streptomycin, 50 μg/ml gentamycin and 2.5 μg/ml amphotericin B. The freshly prepared cell storage medium consisted of 70% (v/v) DMEM, 20% (v/v) FBS and 10% (v/v) dimethylsulfoxide (Sigma, USA).

### Isolation and Culture of OECs

Bovine omasal tissues were obtained from newborn Chinese Holstein calves immediately after they were slaughtered, and then transported to the laboratory in ice-cold DMEM. Serial trypsin digestions were performed to isolate OECs as previously described [23]. Briefly, the tissues were washed several times with ice-cold D-Hank's (balanced salt solution) containing 500 U/ml penicillin, 500 μg/ml streptomycin, 100 μg/ml gentamycin and 5 μg/ml amphotericin B until the solution was clean of any contaminants. Next, the omasal epithelial laminae were minced using a scalpel into approximately 1-cm^2^ pieces. The minced laminae (approximately 20 g, wet weight) were digested in a 250-ml Erlenmeyer flask containing 100 ml of 2.5% trypsin (Amresco, USA) in D-Hank's solution for 1 h at 37°C in a shaking warm-air bath. Next, the digestion solution was discarded and replaced with fresh solution two or three times to remove the stratum corneum epithelia. When a mass of cells with homogeneous morphology emerged and keratinized cells were not predominant, the digestion solution was harvested and replaced with fresh solution, which was approximately every 20 to 30 min, depending on the digestion status. Trypsinization was terminated after cell harvest by adding ice-cold D-Hank's containing 10% FBS into the digestion solution (at a ratio of 1∶1, v/v). After filtration with four layers of 1-mm nylon meshes, the harvested solution was centrifuged at 300×*g* for 5 min at 4°C to remove any residual trypsin from the pellets. Next, the tissue was further digested in 7–8 cycles. The cell yield was assessed using a hemacytometer and the cell viabilities were estimated using trypan blue dye. The cells were resuspended in the growth medium and then seeded at a density of 5×10^4^ cells/ml onto plastic dishes (Corning, USA) coated with collagen I (Gibco). The culture dishes were incubated at 37°C and 5% CO_2_ in a humidified atmosphere and the growth medium was changed every 2 or 3 d according to the growth conditions.

To purify the epithelial cells, confluent cells were first digested using a 0.15% trypsin solution for 10 min. When the fibroblasts detached, and no changes were observed in the OECs, the cells were washed using D-Hank's. Next, the remaining cells were digested for 5–10 min using 0.1% trypsin-0.02% EDTA and seeded onto new culture dishes (1∶2). For cryopreservation, the cells were resuspended in storage medium at a density of 1×10^6^ cells/ml, distributed into cryovials, and stored in liquid nitrogen.

For hematoxylin and eosin staining, the tissues were fixed in 4% formaldehyde solution. After 24 h, the tissues were embedded in paraffin, cut into 5-μm sections and stained with H&E for histological examination using standard procedures.

### Growth Characteristics of OECs

The growth pattern of the OECs *in vitro* was determined by the doubling time of the cells. Cell proliferation was measured using the WST-8 (Boster, China) assay. Briefly, OECs were seeded at 5×10^3^ cells per well in 96-well plates (Corning, USA) in quadruplicates. At specific time points, 10 μl of WST-8 solution was added to each well. Next, the cells were incubated at 37°C for 1 h and the absorbance was determined at a wavelength of 450 nm using a microplate reader (Molecular Devices, USA). The number of viable cells was proportional to the absorbance. The growth and morphology of the cells was assessed using a phase-contrast microscope (Nikon ECLIPSE 50i, Tokyo, Japan).

### Electron Microscopy for the Ultrastructure of OECs

OECs were washed twice with PBS and then fixed with 2.5% glutaraldehyde at 4°C overnight. The cells were rinsed twice with PBS and postfixed with 1% osmium tetroxide for 2 h. After dehydrating the cells in serial ethanol dilutions, the cells were scrapped from their plastic support and embedded in Spurr resin. Ultrathin sections were successively stained with uranyl acetate and alkaline lead citrate for 15 min each and observed using transmission electron microscopy (TEM, JEM-1230, JEOL).

OEC coverslips were first made for scanning electron microscopy (SEM). After fixation and dehydration according to standard procedures for TEM, the specimen was coated with gold-palladium and observed using SEM (Philips, XL30, Holland).

### Immunocytochemical Staining

For cytokeratin staining, the cells were seeded onto laser confocal dishes (Nest, China), and washed 3 times with D-Hank's, fixed with methanol and acetone (v/v) for 10 min at −20°C, and permeabilized with 0.25% PBS-Triton for 10 min. The cells were then incubated in PBS containing 5% normal goat serum to block non-specific protein-protein interactions followed by incubation in primary rabbit anti-cytokeratin 18 antibody (dilution 1∶50, Abcam, HK) and rabbit anti-PEPT1 antibody (dilution 1∶100, Beijing Biosynthesis Biotechnology Co., LTD, China) overnight at 4°C. Subsequently, the cells were incubated in secondary FITC-conjugated goat anti-rabbit IgG antibody (dilution 1∶100, Jackson ImmunoResearch Laboratories, Inc., USA) with DAPI (Sigma, USA). The cells were incubated for 1 h at room temperature in the dark, followed by 3 rinses with PBS for 5 min and visualized using confocal laser microscopy (Leica, TCS SP5, Germany).

### Measurement of PEPT1 mRNA in OECs

Total RNA from bovine omasal tissue and OECs was isolated using ice-cold TRIzol (Invitrogen, USA). The integrity and purity of the RNA were confirmed by assessing the samples using 1% agarose gel electrophoresis and according to the OD ratio at λ260 and λ280 (>1.8) using the NANODROP 2000 Spectrophotometer (Thermo, USA). First strand cDNA was synthesized using the reverse transcription kit (Takara, Shiga, Japan). PEPT1 (NM_001099378) expression was detected using reverse transcription PCR with the following specific primers: (Forward: 5′-TGGCTGGGGAAGTTCAAGAC-3′, Reverse: 5′-TCCTGGCCCTCTTCAAA-3′; Product length: 239 bp), and the glyceraldehyde-6-phosphate dehydrogenase gene (GAPDH, AJ000039) served as an internal control (Primers: Forward 5′-TTGTGATGGGCGTGAACC-3′, Reverse 5′-CCCTCCACGATGCCAAA-3′; Product length: 198 bp). The PCR conditions used were the following: 40 cycles of 5 s at 95°C, 34 s at 60°C and 60 s at 72°C with an initial denaturation of 30 s at 95°C and a final extension of 5 min at 72°C. The amplified PCR products were electrophoresed on a 2% agarose gel and sequenced using a commercial sequencing company (BGI, China). DNA sequences were aligned against known PETP1 sequences from the National Center for Biotechnology Information database.

### Uptake of Gly-Sar into OECs

The uptake of glycylsarcosine (Gly-Sar, model dipeptide) in OECs was investigated as previously described [24], [25]. Hank's balanced salt solution (HBSS: 145 mM NaCl, 3 mM KCl, 1 mM CaCl_2_, 0.5 mM MgCl_2_) containing 5 mM D-glucose and 5 mM HEPES (pH 6.5) was used as the uptake and rinse medium. The OECs were plated at a density of 1×10^5^ cells/cm^2^ in 24-well plates. After confluence (approximately 3 d), the cells were maintained for 7 d to polarize. Next, the cells were observed under an electron microscope, which showed that the cells had differentiated by forming microvilli, desmosomes and tight junctions. On the same day, the OEC monolayers were rinsed twice and preincubated with HBSS for 30 min at 37°C. Uptake was initiated by adding 1 ml of the incubated solution at various pHs (5.0, 5.5, 6.5, 7.5) and for specific time periods (2, 5, 10, 15, 30 min). To study the concentration dependence of Gly-Sar uptake, the cells were incubated with different concentrations (0.5, 1.0, 1.5, 2.5, 5.0 mM) of Gly-Sar at 37°C or 4°C. For the inhibition study, the cells were incubated with different concentrations (0, 0.5, 2.5, 5.0 mM) of methionine-glycine (Met-Gly) in quadruplicates at 37°C. All of the incubations were performed at 37°C for 15 min at pH 6.5 with 2.5 mM Gly-Sar unless noted otherwise. At the end of incubation, the Gly-Sar solution was aspirated, and the cells were quickly washed four times with ice-cold rinse medium (pH 6.5).

To determine the uptake amount of Gly-Sar, the cells were lysed with 0.3 ml of 1% Triton X-100 solution. A portion of the cell lysate was centrifuged at 12000×*g* for 10 min and used to quantify Gly-Sar using high-performance liquid chromatography (HPLC, Agilent, 1100, USA) as described below. The other portion of the lysate was used for protein determination using the BCA protein assay kit (Keygen Biotech, Nanjing, China). The uptake of Gly-Sar was expressed as nmol/mg protein/15 min.

HPLC conditions: The mobile phase was a mixture of buffer A (0.1% trifluoroacetic acid in water) and B (0.1% trifluoroacetic acid in acetonitrile). A gradient elution was run over 10 min, starting from 10% buffer B to 50% buffer B, at a flow rate of 1.0 ml/min. Kromasil 100-5C 18 (4.6 mm×250 mm; Akzo Nobel, Sweden) was used for the analytical column. A sample aliquot (10 μl) was injected into the HPLC system and Gly-Sar was detected at 220 nm.

### Statistical Analysis

Statistical analysis was performed by ANOVA and Duncan's multiple range tests using the SAS software (SAS Institute, 2000). The data were presented as the means ± standard error. The criterion for significance was established at *P*<0.05.

## Results

### Establishment and Growth Characteristics of OECs

**Figure 1:**
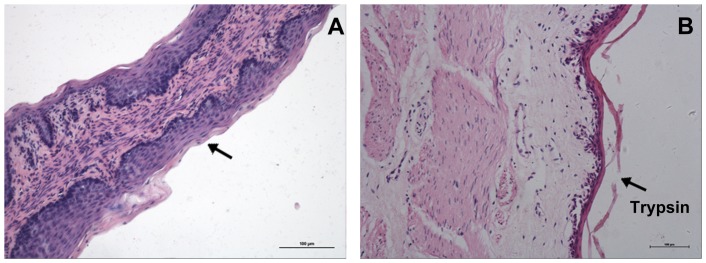
Hematoxylin and eosin (H&E) staining of the omasal wall. A: The omasal wall with intact epithelial strata (arrow) before trypsin treatment. The arrow represents the epithelial strata. B: The omasal wall without epithelial strata after trypsin treatment. The bars represent 100 μm.

**Figure 2:**
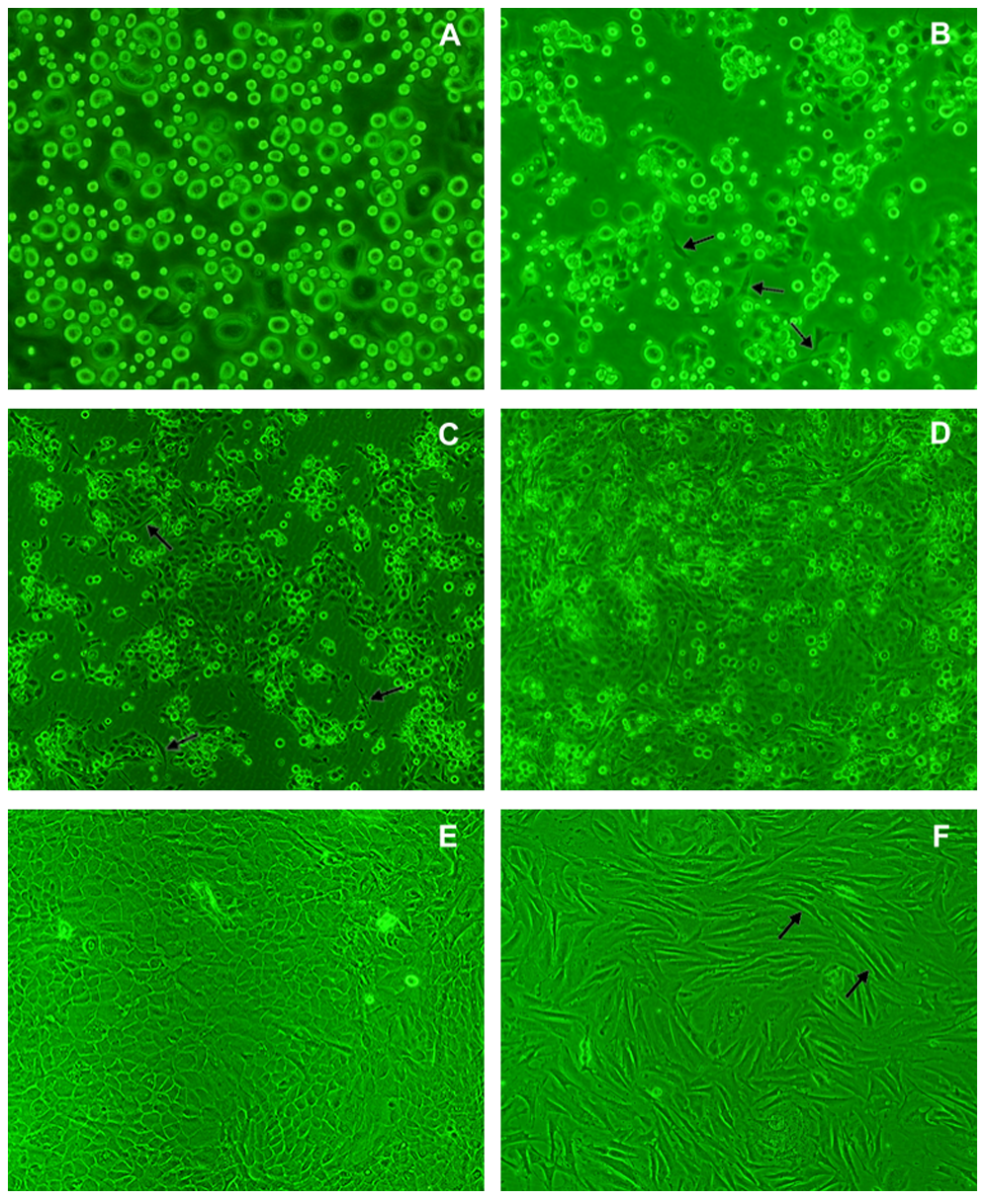
Morphology of isolated omasal epithelial cells at different growth periods. A: Epithelial cell suspensions obtained from newborn calves after enzymatic digestion (×200); B: Most of the omasal epithelial cells adhered to the wall of plastic substratum with small number of fibroblasts after 12 h in culture (×200); C: Cell clusters were observed after 2 d in culture (×100); D: A confluent monolayer formed after approximately one week in culture (×100); E: Purified cultures displayed a cobble-stone-like morphology (×100); F: A confluent monolayer of spindle-shaped, fibroblast-like cells (×100); Fibroblast-like cells are indicated by the arrows.

**Figure 3:**
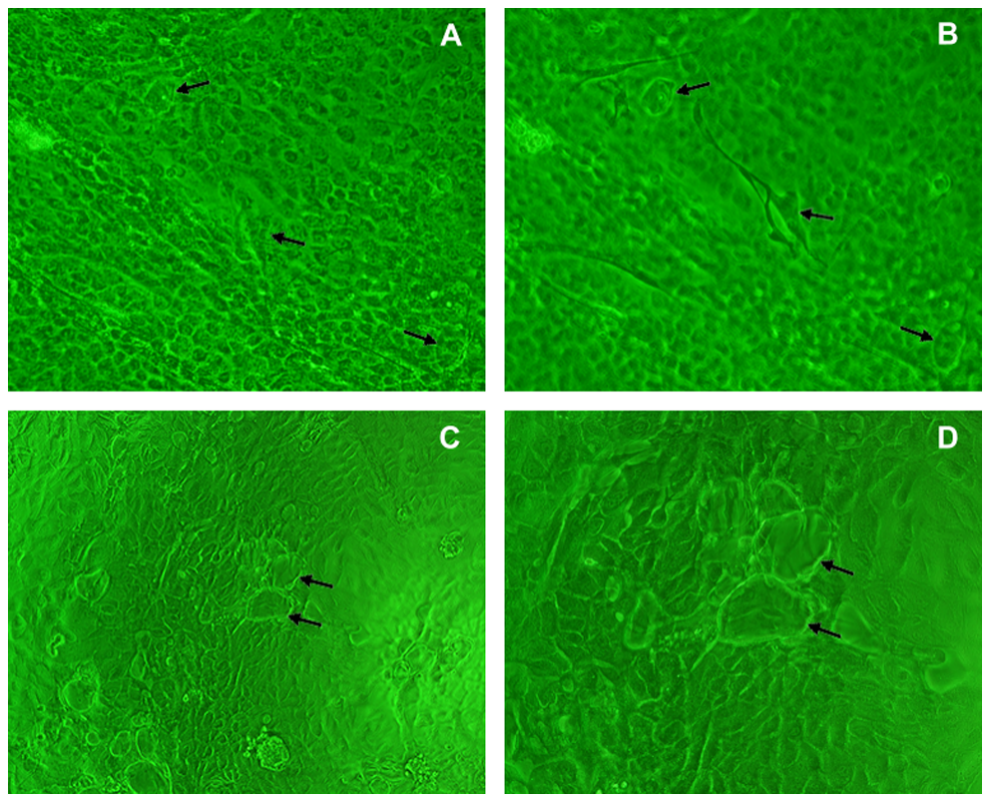
Developed morphology of confluent monolayer of OECs. A: Cuticularized cells originating from post-confluent stage OECs (×200); B: Phase contrast image with the focus set on top of the raised cuticularized cells (×200); the arrows indicate typical cuticularized cells. C (×100) and D (×200): The dome structure (arrows) of the raised layer of cells above the plastic substratum.

After digestion with 2.5% trypsin for 1 to 1.5 h, the corneum was removed. Approximately 5 h later, most of the epithelial cells were successfully isolated from tissues without contamination of the cell sublayers (Figure 1). Viability studies using dye exclusion showed that 90% of the isolated cells were viable (Figure 2-A). The isolated cells adhered to the wall of plastic substratum after 12 h of culture (Figure 2-B). Subsequently, the OECs began to proliferate and formed clusters after 2 to 7 d in culture (Figure 2-C) prior to reaching confluence (Figure 2-D). Fibroblasts were mixed with OECs in primary culture. After digestion with 0.15% trypsin, the fibroblasts first detached from the flask wall and the remaining OECs were then released using a 0.1% trypsin-0.02% EDTA solution. After purification, the OECs displayed a homogeneous ‘cobblestone’ epithelial cell-like morphology with few visible fibroblasts. In addition, there were 2–4 nucleoli in each OEC, and the purified cells showed a clear boundary with a tightly connected pattern on the plastic substratum (Figure 2-E). After freezing and thawing, 90% of the OECs were viable and exhibited normal epithelial morphology. Several days after the formation of the cell monolayer, cuticularized cells emerged above the OECs (Figure 3-A, B) and the monolayer demonstrated dome-shaped structures (Figure 3-C, D).

**Figure 4:**
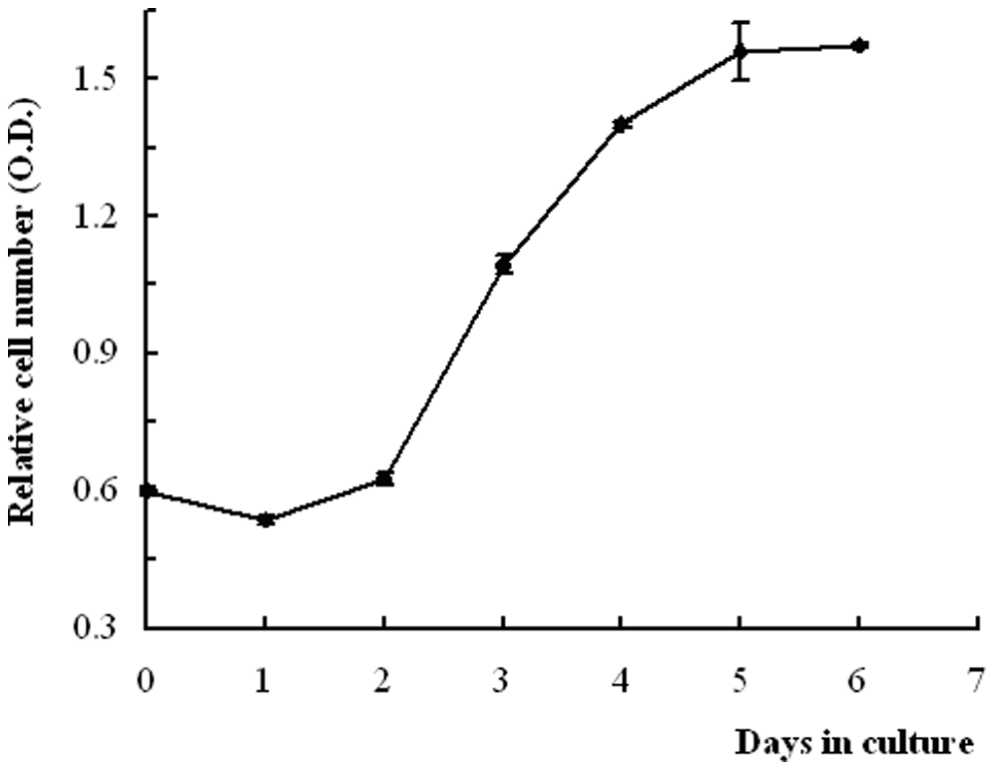
Growth curve of OECs in culture. The log phase started after day 2 of the lag phase with a sharper inclination and cells entering the logarithmic growth phase on day 5. N = 4.

The growth pattern of the OECs was plotted in Figure 4. After an initial delay of 2 d, the cells entered the logarithmic growth phase (day 2–5), and then the cells grew slowly and reached the plateau phase. OECs demonstrated a stable growing ability with a population doubling time of 62 h (Figure 4).

### Ultrastructure of OECs

**Figure 5:**
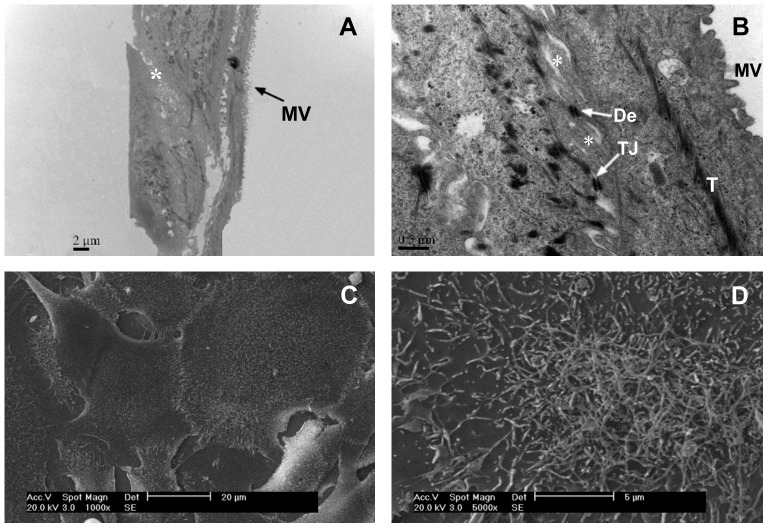
Transmission electron micrograph (TEM, A and B) and scanning electron micrograph (SEM, C and D) of OEC monolayers. A: A polarized monolayer was established with apical microvilli (MV) and a basal lamina on the plastic substratum; B: Connections between neighboring cells via desmosomes (De), tight junctions (TJ) and basolateral membrane infoldings (*) were visible. Tonofilaments (T) were also observed in the OECs. C and D: SEM observations revealed numerous microvilli-like structures on the surface of the OEC monolayer.

After a monolayer of OECs was formed at 7 d in culture, the ultrastructure of the OEC monolayers was observed using TEM and SEM (Figure 5). A polarized monolayer was observed with apical microvilli and a basal lamina on the plastic substratum. Microvillus is a characteristic of luminal intestinal epithelial cells [26]. In addition, desmosomes, tonofilaments, tight junctions and basolateral membrane infoldings were observed between neighboring cells of the confluent cell monolayers. OEC monolayers of this type were used in the transport studies described below.

### Expression of Cytoskeleton 18 and PEPT1

**Figure 6:**
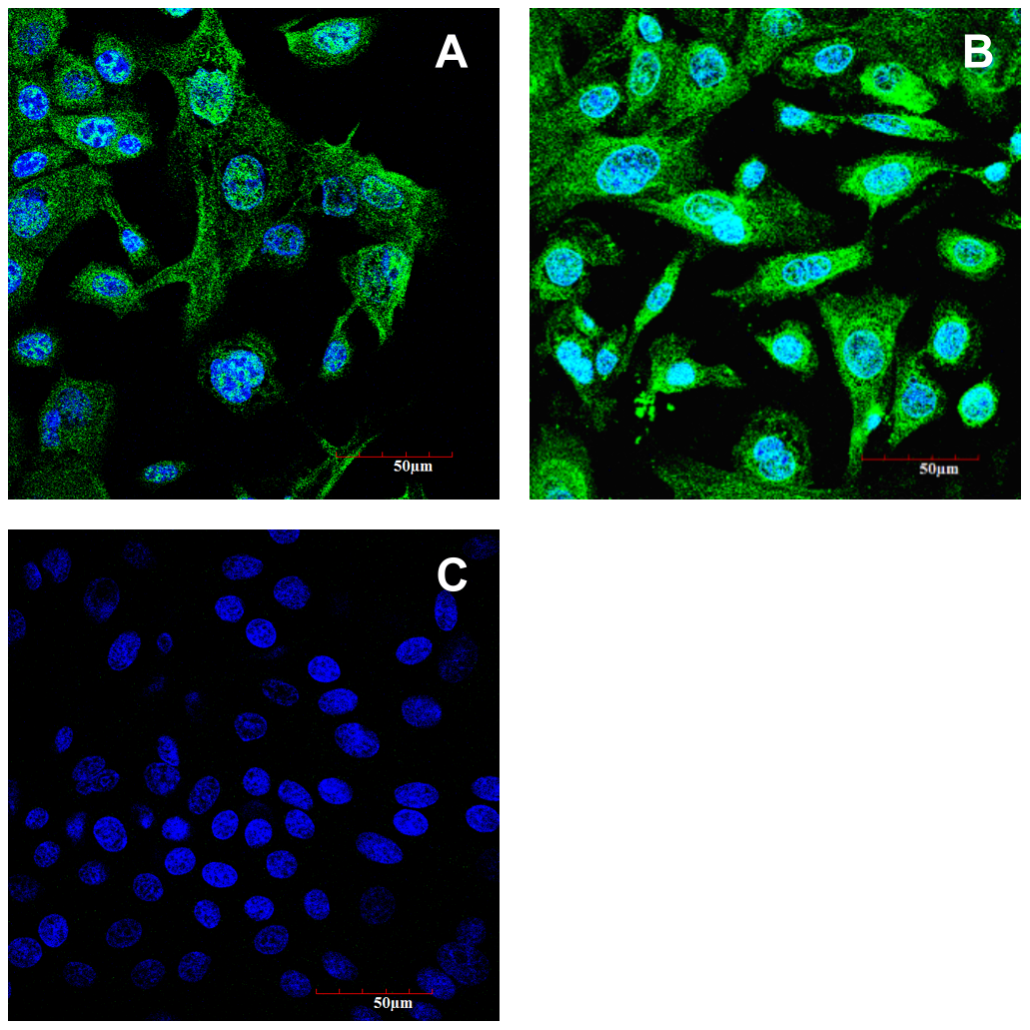
Immunofluorescence staining of OECs. Fluorescent images of OECs stained for cytokeratin 18 (A), PEPT1 (B) and negative control using the secondary antibody only (C). Nuclei were stained with DAPI.

OECs were stained to examine the expression of cytoskeleton 18, an intermediate filament protein and a marker of epithelial cells [27]–[29]. The cells exhibited a strong immunopositive staining for cytoskeleton 18 (Figure 6-A). PEPT1 antigen expression was detectable by immunocytochemistry in cytoplasm and nuclei of OECs (Figure 6-B). A negative control using only the secondary antibody revealed no specific staining in the cells (Figure 6-C).

**Figure 7:**
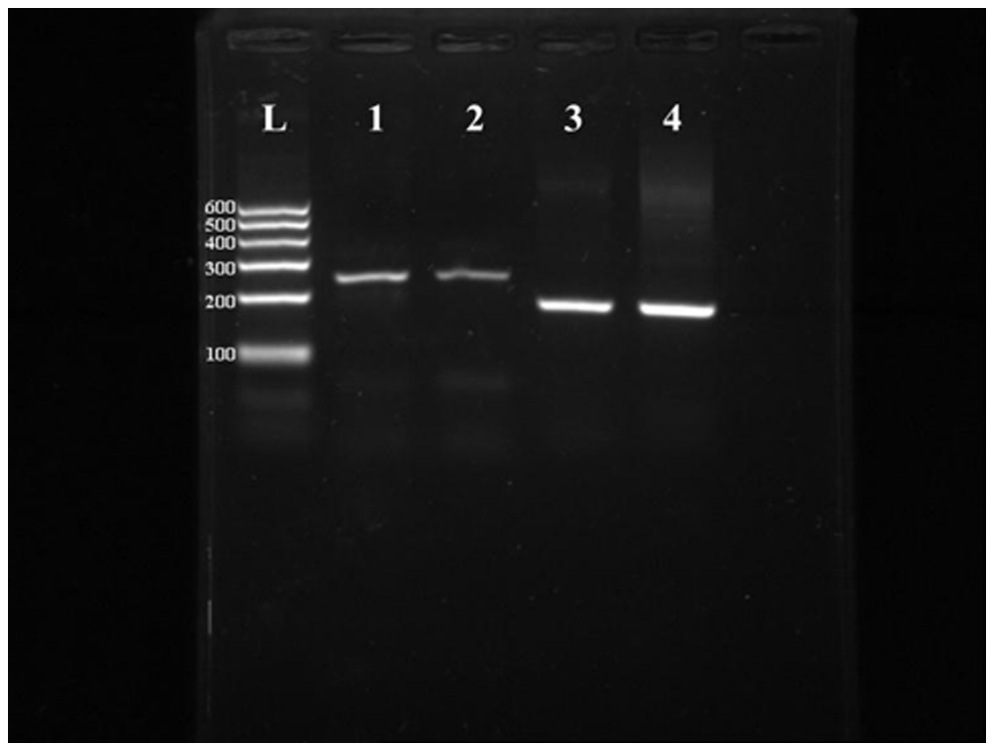
Reverse transcription PCR of PEPT1 gene expression in omasal epithelial tissue and OECs. Lane L: 100 bp DNA ladder; lane 1: Omasal epithelial tissue; lane 2: OECs; lane 3 and 4: Loading control of the expression of the housekeeping gene glyceraldehydes-3-phosphate dehydrogenase (GAPDH) of tissue (3) and omasal epithelial cells (4).

PEPT1 mRNA was expressed in both the epithelial tissue and epithelial cells (Figure 7). Sequencing of the amplified PCR products showed a 100% match to known bovine mitochondrial DNA sequences in GenBank, which further revealed the epithelial origin of the isolated cells and suggested that these cells could be used to study the transport properties of PEPT1.

### Effects of pH and Time on Gly-Sar Uptake by OECs

**Figure 8:**
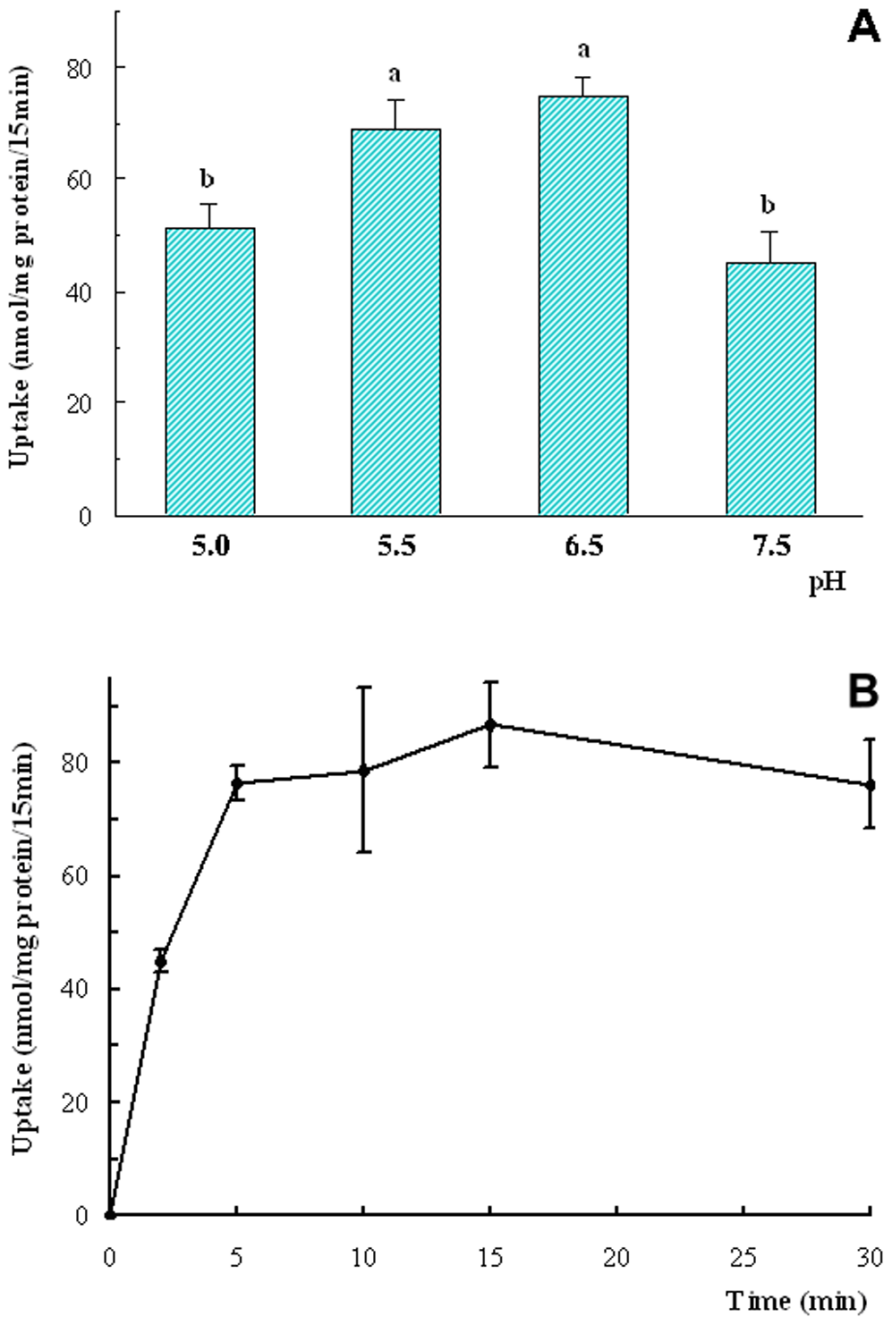
The effects of pH and time on Gly-Sar uptake by OECs. A: Uptake of Gly-Sar at various pHs for 15 min. B: The uptake of Gly-Sar at various time points (min) under pH 6.5. N = 4. Values with different letter (a, b) indicate significant difference (*P*<0.05).

Isolated OECs showed a significant uptake of Gly-Sar dipeptide (Figure 8) and the uptake was affected by extracellular pH. The uptake was also significantly higher at pH 5.5 and 6.5 than at pH 5.0 and 7.5 (*P*<0.05, Figure 8-A), indicating that the transport process was pH-dependent. Thus, an extracellular pH of 6.5 was used in subsequent experiments. The optimal time of incubation for the uptake of Gly-Sar was also determined in OECs (Figure 8-B). The uptake of Gly-Sar by OECs increased as the incubation time increased and reached a plateau between 5 and 15 min. Thus, in all of the subsequent experiments, the time of incubation for the uptake measurements was 15 min to ensure that the transporter was saturated.

### Effects of Substrate Concentration, Temperature and Competitive Inhibitor on Gly-Sar Uptake by OECs

**Figure 9:**
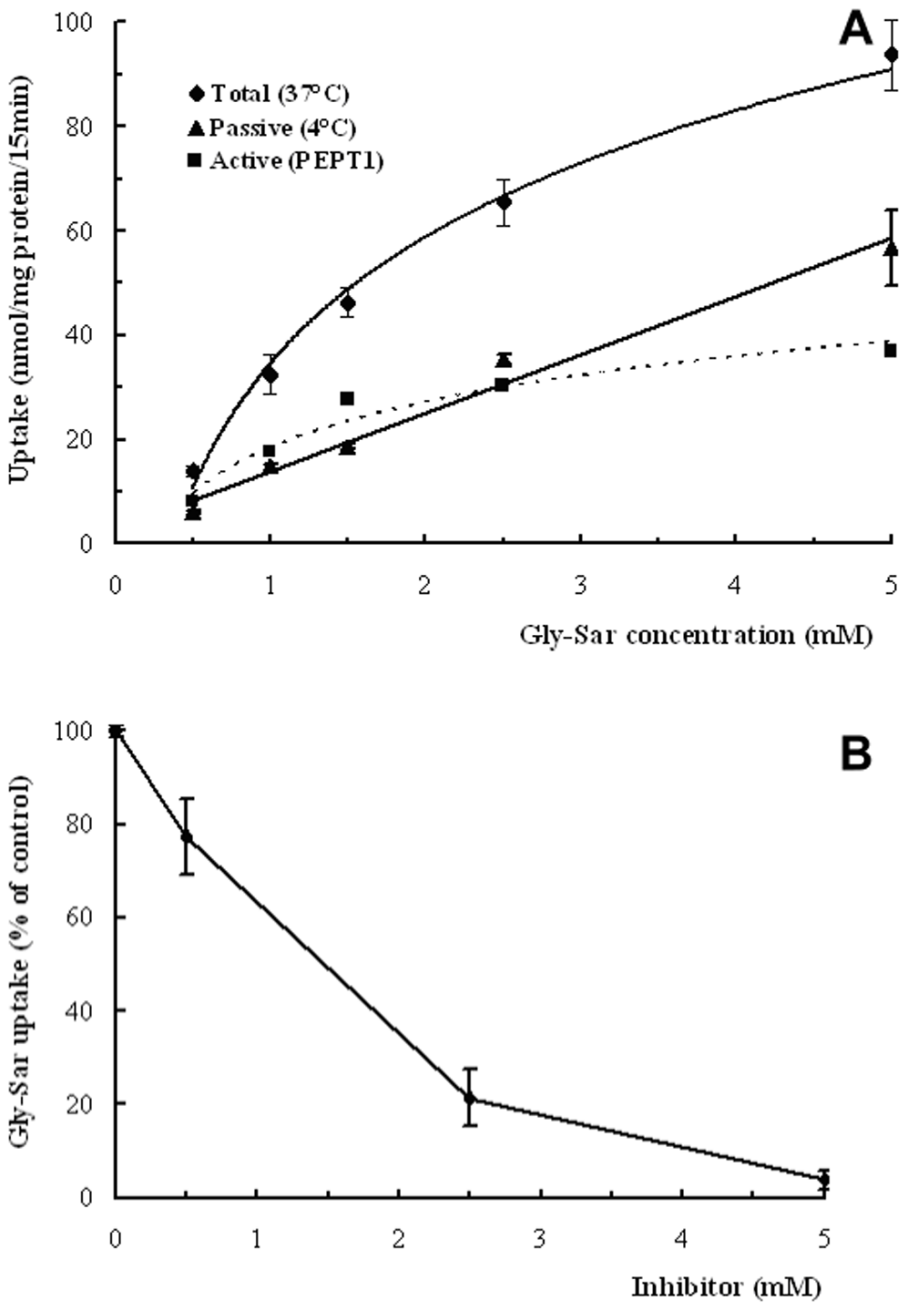
Effects of substrate concentration, temperature and competitive inhibitor on Gly-Sar uptake by OECs. A: OEC monolayers were incubated with various concentrations (0–5 mM) of Gly-Sar at 37°C (⧫) or 4°C (▴). Active (▪) uptake via PEPT1 was estimated according to the difference between 37°C and 4°C. B: The uptake of 0.5 mM Gly-Sar was measured in the absence or presence of increasing concentrations of methionine-glycine (0–5 mM). Uptake of Gly-Sar measured in the absence of the inhibitor dipeptide was set as 100%. N = 4.

The effects of temperature and Gly-Sar concentration on the uptake of Gly-Sar were also examined in OECs. The uptake of Gly-Sar was significantly higher at 37°C compared to 4°C (*P*<0.05). In addition, the uptake at 4°C was directly proportional to the concentration of Gly-Sar and increased linearly without a plateau (Figure 9-A). The effect of temperature on uptake was via PEPT1 rather than a passive route. In addition, active uptake via PEPT1 was estimated by the difference between the total uptake at 37°C and passive uptake at 4°C [13]. Even at a low substrate concentrations (<2.5 mM) in the medium, the uptake at 37°C was higher compared to 4°C, indicating that a transporter-mediated process contributed more to the absorption at the low concentrations. Furthermore, the transporter-mediated uptake was saturated with 2.5 mM Gly-Sar in the medium. When the concentrations of Gly-Sar were greater than 2.5 mM, the uptake at 37°C was similar to that at 4°C. In addition, 2.5 mM Gly-Sar was used in subsequent competition inhibitory experiments, where Met-Gly inhibited the uptake of Gly-Sar (Figure 9-B). Taken together, these results suggested that the same carrier proteins transported both Gly-Sar and Met-Gly.

## Discussion

### Establishment and Characterization of OECs

As early as newborn age, the epithelium of the ruminant omasum displays an intact morphological structure with complete functions [22]. In this study, we successfully developed a method to isolate OECs from newborn calves according to previously described procedures used to isolate epithelial cells of the rumen [23]. Morphological examination and PEPT1 mRNA and protein expression in the omasal epithelium of newborn calves that did not receive the creep diet confirmed that newborn calves were structurally and functionally equipped to absorb small peptides similar to adults. We also tried to isolate OECs from adult dairy cows, but were unfortunately not successful because the cells were highly differentiated and difficult to purify, proliferate and subculture. Because of the rapid self-renewing rate of calf OECs, the cells had a higher proliferation rate compared to adult OECs. In addition, it is much easier to obtain clean tissues from newborn calves because their gastrointestinal tracts were not contaminated with feeds and microorganisms.

The stratum corneum, which primarily consists of dead cells, acts as a barrier to nutritional absorption [30] and must be first removed to isolate the epithelia that lie underneath. Previous studies have shown that primary ruminal epithelial cells may be isolated from the rumen using dissociation solutions containing various concentrations of trypsin. In our study, five different protocols of enzymatic digestion were examined (0.25, 0.625, 1.25, 2.5, 5% trypsin) to optimize the OEC isolation. We found that 2.5% trypsin was the most effective in the isolation, viability and adherence of OECs *in vitro* (data not shown). It was also difficult to isolate the cells due to the sensitivity of the cells in response to trypsin; for example, higher concentrations of trypsin were harmful to the cells and lower concentrations were not sufficiently effective in isolating the cells. In addition, the omasal lamina needed to be cut into 1-cm^2^ pieces rather than mashed to avoid the contamination of muscle or other cells from the deeper strata underlying the epithelium. The epithelial strata origin of our isolated OECs was confirmed using H&E staining of the omasum from which the cells were isolated.

In this study, fibroblast and epithelial cells were separated according to their different sensitivities to trypsin, as previously reported [28], [31]. Previously, a variety of methods were used to inhibit the growth of fibroblasts; however, it was impossible to completely eliminate these cells using these methods [32]. After a series of purification steps, we were able to eliminate the majority of contaminating non-epithelial cells (mostly fibroblasts); however, a small number of fibroblasts (less than 10% of cells in culture) were still present with the OECs. Previous studies indicated that the presence of non-epithelial cells in the cultures may have beneficial effects for epithelial cell growth and viability [33].

In this study, our isolated OECs exhibited a typical cobblestone morphology with microvilli, which were also observed on the apical surface of other isolated bovine intestinal epithelial cell lines [34], [35] and primary cells [36], [37]. In addition, our cells also identified the omasal epithelium as assessed using electron microscopy, which revealed the presence of intercellular junctions (desmosomes and tight junctions) and tonofilaments, which were also observed in primary epithelial cells from the bovine rumen [23] and colon epithelium [37]. Furthermore, the cells possessed the capacity to continuously proliferate to form a confluent monolayer, and express PEPT1 and cytokeratin 18. These properties revealed the epithelial nature of these cells. Moreover, the growth of OECs on the plastic substratum revealed a population doubling time of 62 h and exhibited an S-shaped growth curve (Figure 4), representing the characteristics of normal a non-transformed phenotype. Thus, it was better to use these cells for studies 5 d after seeding the cells when the confluent monolayer had been well-formed.

In OECs primary cultures, cuticularized cells emerged in most areas outside of the monolayer, suggesting that our isolated OECs underwent differentiation and maturation *in vitro* similar to the epithelium *in vivo*. Moreover, the OECs exhibited dome-like structures at post-confluent stages, which appeared sporadically when the cells grew on a plastic substratum coated with collagen. This structure has been reported to develop due to an accumulation of fluid underneath the epithelial cell layer [38]. The formation of spontaneous dome structures in the OECs suggested that these cells underwent differentiation and secreted basement membrane components similar to mammary epithelial cells reported in previous studies [27], [29].

### Study of Small Peptide Absorption by Using the OEC Model

PEPT1 is an H^+^-coupled peptide transport protein. Our study showed that OECs express PEPT1 mRNA and take up Gly-Sar in a pH-dependent manner at an optimal pH of 6.5. These observations indicated that PEPT1 might be involved in small peptide uptake in OECs, and this uptake was efficient at physiological pH conditions in the forestomach, which is approximately pH 6.0 in dairy cows [39]. It has also been reported that the peptides are absorbed by the gastrointestinal tract via two routes of active (transporter-mediated) and passive (paracellular movement and cell-penetrating peptides) mechanisms. The active route is saturable by the absorbed peptides, whereas the passive route is not saturable [7], [13]. In addition, the active route is temperature-sensitive whereas the passive route is not temperature-sensitive. In this study, the absorption of Gly-Sar in cells was affected by temperature and was saturable by the substrate, indicating that the absorption was partly mediated by peptide transporters. However, our data also showed that Gly-Sar was absorbed by OECs via the passive route because the uptake occurred at 4°C [13], and the absorption exhibited a linear increase, corresponding with an increase in concentration. Our kinetic assay also indicated that the peptide transporter demonstrated a high affinity to the substrate and functioned efficiently at low concentrations.

Our study further showed that the peptide transport system exhibits multiple substrate specificities because Met-Gly was able to efficiently inhibit Gly-Sar uptake. Met-Gly has been shown to be a substrate of PEPT1 in a transfected mammalian cell line [17] and in Chinese hamster ovary cells [18], which further support the potential involvement of PEPT1 in the uptake of these peptides in OECs. The low concentrations of Met-Gly required to inhibit 50% of Gly-Sar uptake also indicated that the transporter system demonstrated a high affinity for Met-Gly. Finally, these studies demonstrated that our OEC model was a suitable system to study the peptide transport process in the forestomach of ruminant animals.

In conclusion, we successfully established and maintained the primary culture of OECs from newborn dairy calves. The cultured cells retained the morphological and immunological characteristics of epithelial cells. Moreover, OECs could also take up small peptides *in vitro*, and the uptake was dependent on the substrate concentration, temperature and protein and may be inhibited using competitors. We also observed that PEPT1 may be responsible for the absorption of small peptides in OECs. OECs can be used as an important model for the *in vitro* study of the absorption mechanisms of various nutrients, including small peptides in the bovine forestomach.
